# TNF-*α* inhibitor reduces drug-resistance to anti-PD-1: A mathematical model

**DOI:** 10.1371/journal.pone.0231499

**Published:** 2020-04-20

**Authors:** Xiulan Lai, Wenrui Hao, Avner Friedman

**Affiliations:** 1 Institute for Mathematical Sciences, Renmin University of China, Beijing, P. R. China; 2 Department of Mathematics, Pennsylvania State University, State College, PA, United States of America; 3 Mathematical Bioscience Institute & Department of Mathematics, Ohio State University, Columbus, OH, United States of America; University of California Irvine, UNITED STATES

## Abstract

Drug resistance is a primary obstacle in cancer treatment. In many patients who at first respond well to treatment, relapse occurs later on. Various mechanisms have been explored to explain drug resistance in specific cancers and for specific drugs. In this paper, we consider resistance to anti-PD-1, a drug that enhances the activity of anti-cancer T cells. Based on results in experimental melanoma, it is shown, by a mathematical model, that resistances to anti-PD-1 can be significantly reduced by combining it with anti-TNF-*α*. The model is used to simulate the efficacy of the combined therapy with different range of doses, different initial tumor volume, and different schedules. In particular, it is shown that under a course of treatment with 3-week cycles where each drug is injected in the first day of either week 1 or week 2, injecting anti-TNF-*α* one week after anti-PD-1 is the most effective schedule in reducing tumor volume.

## Introduction

Drug resistance is one of the primary causes for suboptimal outcomes in cancer therapy [1]. Patients respond well at first, but relapse occurs for many cancer patients [2]. One common resistance is associated with the ATP-binding cassette (ABC) transporters. These protein pumps act to protect cells by ejecting variety of toxins, and they are overexpressed during anti-cancer treatment with chemotherapy and targeted therapy [1–3].

Other mechanisms of drug resistance are associated with mutations and epigenetic changes [4]. There are many mathematical models of drug resistance, particularly resistance to chemotherapy; e.g., a model of evolution of resistance [5], and a model of resistance associated with symmetric/asymmetric division of stem cells [6]. Article [7] reviews the mathematical models up to 2011, and very recent reviews are found in [8, 9]. Recent models considered multi-mutations in drug resistance for specific cases [10], and optimal therapy design to reduce drug resistance [11]. A list of the mathematical and computational methods used to simulate models of drug resistance are given in [12, 13]; in particular, articles [4, 14] use PDE models, as do the recent papers [15, 16] and the present one.

There are different mechanisms of resistance to different drugs. In this paper we consider resistance to the immunotherapy drug anti-PD-1, and show that injection of TNF-*α* will reduce the resistance.

PD-1 is a checkpoint on T cells. Its ligand PD-L1 is expressed on both T cells and cancer cells. The formation of the complex PD-1-PD-L1 initiates a signaling cascade that results in blocking the anti-cancer activity of T cells. PD-1 blockade by anti-PD-1 drugs, is currently increasing used in the treatment of cancers.

Internal mechanisms of drug resistance to anti-PD-1 include the following:

- **B2M mutation:** Loss of b2-microglobulin (B2M) expression results in impaired cell surface expression of HLA class I (MHC class I), which in turn impairs antigen presentation to cytotoxic T cells, and thereby leads to anti-PD-1 resistance [17–20].
- **JAK-1/JAK-2 mutation:** INF-*γ* released by T cells activates the signalling pathway. JAK1/JAK2-STAT1/STAT2/STAT3-IRF1, which leads to upregulation of PD-L1 on cancer cells [21]. Acquired resistance to PD-1 blockade immunotherapy in patients with melanoma was associated with defects in the pathways involved in interferon-receptor signaling, such as mutation of interferon-receptor-associated Janus kinase 1 (JAK1) or Janus kinase 2 (JAK2) [17, 20, 22]. Inability to respond to IFN-*γ* leads to reduced expression of PD-L1 on cancer and hence to reduced effectiveness of anti-PD-1.
- **Loss of neoantigen:** Genetic mutations that make cells become cancer cells may result in production of proteins that immune system can recognize as antigen; these are called neoantigens. Anti-PD-1 treatment may cause mutations in cancer cells that result in loss of neoantigens, and, correspondingly, to a decrease in immune response, including decreases in the number of active anti-cancer T cells, and hence a decrease in the effectiveness of anti-PD-1 [23].

Another form of drug resistance is associated with a change undergoing in the anti-cancer CD8^+^ T cells (cytotoxic T cells, CTLs):

- **TIM-3 checkpoint:** Under anti-PD-1 treatment, T-cell immunoglobulin mucin-3 (TIM-3) is upregulated on T cells [24]. Its ligand, Galectin-9 (Gal-9) is expressed on cancer cells, and the complex formed by the checkpoint TIM-3 and its ligand Gal-9 induces apoptosis of Th1 cells [25, 26], and, consequently, a reduction in CD8^+^ T cells whose proliferation depends on Th1-secreted IL-2.

Recent approaches to overcome anti-PD-1 drug resistance focus on combination strategies [27]. A prime example is the combination of anti-PD-1 and anti-CTL-4 [28–31].

TNF-*α* is a pleiotropic cytokine that is involved in diverse functions. In immune response to cancer it acts as immunosuppressive [32]. TNF-*α* has been shown to enhance the expression of PD-L1 on CD8^+^ T cells in cancer [33], including melanoma [34]. TNF-*α* elicits an increase in TIM-3+ CD8^+^ T cells, and anti-PD-1 triggers TIM-3 expression in TNF-*α* dependent manner [32]. Hence blockade of TNF-*α* overcomes resistance to anti-PD-1 by reducing TIM-3 and PD-L1 expression.

For simplicity, we shall combine CD4^+^ Th1 cells with CD8^+^ T cells, since they play similar roles in the response to anti-PD-1 drug resistance, and refer to the combined populaltion of T cells as cytotoxic lymphocytes (CTL).

In the present paper we develop a mathematical model of combination therapy with anti-PD-1 and anti-TNF-*α*. The model will be used to simulate the efficacy of anti-PD-1 under different amount of anti-TNF-*α*, and under different schedules of treatment.

**Fig 1:**
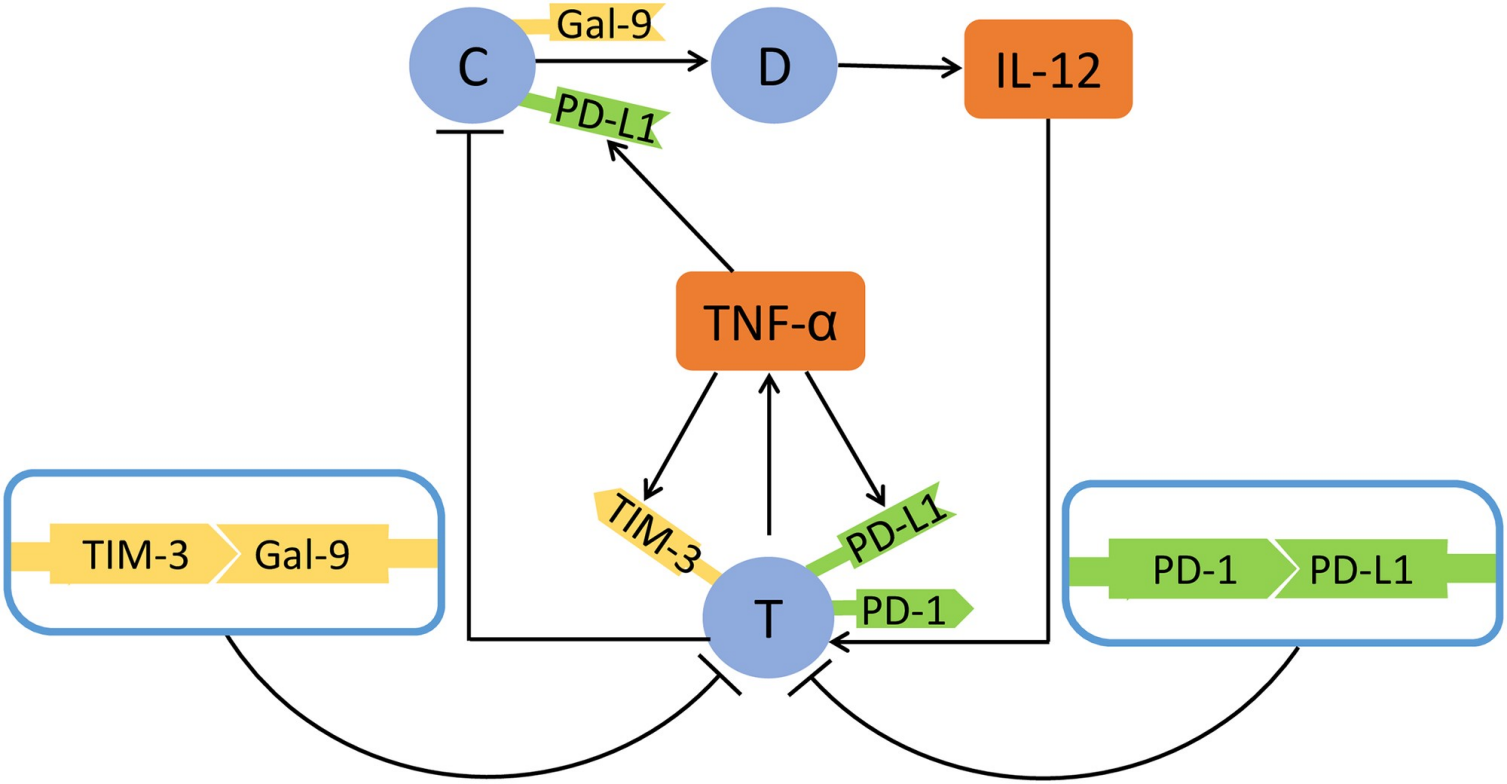
Interaction of immune cells with cancer cells. Sharp arrows indicate proliferation/activation, blocked arrows indicate killing/blocking. C: cancer cells, D: dendritic cells, *T*: activated CTL cells. T cells express PD-1, PD-L1, TIM-3; cancer cells express PD-L1 and Gal-9.

The model includes the densities of cancer cells (*C*), dendritic cells (*D*) and CTL cells (*T*), the concentrations of cytokines TNF-*α* and IL-12, and the proteins PD-1, PD-L1, TIM-3 and Gal-9. Resistance to anti-PD-1 treatment is modeled by upregulation of TIM-3 [24]. The model is based on the network shown in Fig 1, and will be represented by a system of partial differential equations.

## The model equations

The list of variables is given in Table 1 in unit of g/cm^3^; the unit of time is taken to be 1 day. We assume that the total density of cells within three tumor remains constant in space and time:

$$\begin{array}{c} D+T+C=\text{constant}. \end{array}$$ (1)

**Table 1:** List of variables (in units of g/cm^3^).

Notation	Description
*C*	density of cancer cells
*D*	density of dendritic cells
*T*	density of activated CTL cells
*T*_*α*_	TNF-*α* concentration
*I*_12_	IL-12 concentration
*P*	PD-1 concentration
*L*	PD-L1 concentration
*Q*_1_	PD-1-PD-L1 concentration
*Q*_2_	TIM-3-Gal-9 concentration
*T*_*M*_	TIM-3 concentration
*G*	Gal-9 concentration
*A*	anti-PD-1 (e.g. nivolumab)
*B*	anti-TNF-*α* (e.g. infliximab)

Under the assumption (1), proliferation of cancer cells and immigration of immune cells into the tumor give rise to internal pressure among the cells, which results in cells movement. We assume that all the cells move with the same velocity, **u**; **u** depends on space and time and will be taken in units of cm/day. We also assume that all the cells undergo dispersion (i.e., diffusion), and that all the cytokines and anti-tumor drugs are also diffusing within the tumor.

### Equations for DCs (*D*)

By necrotic cancer cells we mean cancer cells undergoing the process of necrosis. Necrotic cancer cells release HMGB-1 [35]. Dendritic cells are activated by HMGB-1 [36, 37], and we assume that the density of HMGB-1 is proportional to the density of cancer cells. Hence the dynamics of activated dendritic cells is given by

$$\begin{array}{c} \begin{array}{c} \frac{\partial D}{\partial t}+\underset{\text{velocity}}{\underset{︸}{\nabla \cdot (uD)}}-\underset{\text{difusion}}{\underset{︸}{\delta_{D}\nabla^{2}D}}=\underset{\text{activation}\;\text{by}\;\text{necrotic}\;\text{cells}}{\underset{︸}{\lambda_{DC}{\hat{D}}_{0}\frac{C}{K_{C}+C}}}-\underset{\text{death}}{\underset{︸}{d_{D}D}}, \end{array} \end{array}$$ (2)

where ${\hat{D}}_{0}$ is the density of immature dendritic cells which we assume to be constant, *δ*_*D*_ is the dispersion (or diffusion) coefficients of DCs, and *d*_*D*_ is the death rate of DCs.

### Equations for CTL cells (*T*)

Inactive T cells are activated by IL-12 [38, 39]. The processes of activation and proliferation of *T* cells are inhibited by the complexes PD-1-PD-L1 (*Q*_1_) and TIM-3-Gal-9 (*Q*_2_). We represents these inhibitions by factors $\frac{1}{1+Q_{1}/K_{TQ_{1}}^{\prime }}$ and $\frac{1}{1+Q_{2}/K_{TQ_{2}}^{\prime }}$, respectively, and take (see Eq (10)) $\frac{1}{1+Q_{1}/K_{TQ_{1}}^{\prime }}=\frac{1}{1+PL/K_{TQ_{1}}}$ and, similarly, $\frac{1}{1+Q_{2}/K_{TQ_{2}}^{\prime }}=\frac{1}{1+T_{M}G/K_{TQ_{2}}}$. Hence *T* satisfies the following equation:

$$\begin{array}{ccc} \frac{\partial T}{\partial t}+\nabla \cdot \left(uT\right)-\delta_{T}\nabla^{2}T & = & \underset{\text{activation}\;\text{by}\;\text{IL}-12}{\underset{︸}{\lambda_{TI_{12}}{\hat{T}}_{0}\cdot \frac{I_{12}}{K_{I_{12}}+I_{12}}}}\cdot \underset{\text{inhibition}\;\text{by}\;\text{PD}-1-\text{PD}-L1}{\underset{︸}{\frac{1}{1+PL/K_{TQ_{1}}}}}\\ & \; & \times \underset{\text{inhibition}\;\text{by}\;\text{TIM}-3-\text{Gal}-9}{\underset{︸}{\frac{1}{1+T_{M}G/K_{TQ_{2}}}}}-\underset{\text{death}}{\underset{︸}{d_{T}T}} \end{array}$$ (3)

where ${\hat{T}}_{0}$ is the density of the inactive T cells.

### Equation for tumor cells (*C*)

We assume a logistic growth for cancer cells with carrying capacity (*C*_*M*_) in order to account for competition for space among these cells. Cancer cells are killed by T cells. The equation for *C* takes the form:

$$\begin{array}{c} \begin{array}{cc} \frac{\partial C}{\partial t}+\nabla \cdot \left(uC\right)-\delta_{C}\nabla^{2}C= & \underset{\text{proliferation}}{\underset{︸}{\lambda_{C}C(1-\frac{C}{C_{M}})}}-\underset{\text{killing}\;\text{by}T\text{cells}}{\underset{︸}{\eta TC}}-\underset{\text{death}}{\underset{︸}{d_{C}C,}} \end{array} \end{array}$$ (4)

where *η* are the killing rates of cancer cells by *T*, and *d*_*C*_ is the natural death rate of cancer cells (by apoptosis).

### Equation for IL-12 (*I*_12_)

The proinflammatory cytokine IL-12 is secreted by activated DCs [38, 39], so that

$$\begin{array}{ccc} \frac{\partial I_{12}}{\partial t}-\delta_{I_{12}}\nabla^{2}I_{12} & = & \underset{\text{production}\;\text{by}\;\text{DCs}}{\underset{︸}{\lambda_{I_{12}D}D}}-\underset{\text{degradation}}{\underset{︸}{d_{I_{12}}I_{12}.}} \end{array}$$ (5)

Since the diffusion coefficients of proteins are very large compared to those of cells, their advection velocity may be neglected.

### Equation for TNF-*α* (*T*_*α*_)

Macrophages are attracted to the tumor, and they produce TNF-*α* [40]. For simplicity, we do not include macrophages in the model, and represent their TNF-*α* contribution as a source $A_{T_{\alpha }}$. The cytokine TNF-*α* is also secreted by T cells, so that

$$\begin{array}{ccc} \frac{\partial T_{\alpha }}{\partial t}-\delta_{T_{\alpha }}\nabla^{2}T_{\alpha } & = & A_{T_{\alpha }}+\underset{\text{production}\;\text{by}T\text{cells}}{\underset{︸}{\lambda_{T_{\alpha }T}T}}-\mu_{T_{\alpha }B}T_{\alpha }B-\underset{\text{degradation}}{\underset{︸}{d_{T_{\alpha }}T_{\alpha },}} \end{array}$$ (6)

where *B* is the TNF-*α* inhibitor.

### Equation for PD-1 (*P*), PD-L1 (*L*) and PD-1-PD-L1 (*Q*_1_)

PD-1 is expressed on the surface of activated CD8^+^ T cells [41, 42]. We denote by *ρ*_*T*_ the mass of all the PD-1 in one T cell divided by the mass of a T cell, so that

$$\begin{array}{c} P=\rho_{P}T. \end{array}$$ (7)

PD-L1 is expressed on the surface of activated CD8^+^ T cells [42] and cancer cells [42, 43]. We denote by *ρ*_*L*_ the mass of all the PD-L1 in one T cell divided by the mass of the cell, and by *ρ*_*L*_ *ε*_*C*_ the mass of all the PD-L1 in one cancer cell divided by the mass of the cell, so that

$$\begin{array}{c} L=\rho_{L}\left(T+\varepsilon_{C}C\right). \end{array}$$

The expression of PD-L1 is upregulated by TNF-*α* [33], so that

$$\begin{array}{c} L=\rho_{L}\left(T+\varepsilon_{C}C\right)(1+\alpha_{L}\frac{T_{\alpha }}{K_{T_{\alpha }}+T_{\alpha }}). \end{array}$$ (8)

The coefficient *ρ*_*P*_ is constant when no anti-PD-1 drug is administered. And in this case, to a change in *T*, given by $\frac{\partial T}{\partial t}$, there corresponds a change of *P*, given by $\rho_{P}\frac{\partial T}{\partial t}$. For the same reason, ∇ · (**u***P*) = *ρ_P_* ∇ ·(**u***P*) and ∇^2^ *P* = *ρ*_*P*_∇^2^ *P* when no anti-PD-1 drug is injected. Hence, *P* satisfies the equation

$$\begin{array}{c} \begin{array}{cc} \frac{\partial P}{\partial t}+\nabla \cdot \left(uP\right)-\delta_{T}\nabla^{2}P & =\rho_{P}[\frac{\partial T}{\partial t}+\nabla \cdot \left(uT\right)-\delta_{T}\nabla^{2}T]. \end{array} \end{array}$$

Recalling Eq (3) for *T*, we get

$$\begin{array}{c} \begin{array}{cc} \frac{\partial P}{\partial t}+\nabla \cdot \left(uP\right)-\delta_{T}\nabla^{2}P= & \rho_{P}[\text{RHS}\;\text{of}\;\text{Eq.}\;\text{(}3\text{)}]. \end{array} \end{array}$$

When anti-PD-1 drug (*A*) is applied, PD-1 is depleted by *A*, and the ratio $\frac{P}{T}$ may change. In order to include in the model both cases of with and without anti-PD-1, we replace *ρ*_*P*_ in the previous equation by $\frac{P}{T}$. Hence,

$$\begin{array}{c} \begin{array}{cc} \frac{\partial P}{\partial t}+\nabla \cdot \left(uP\right)-\delta_{T}\nabla^{2}P= & \frac{P}{T}[\text{RHS}\;\text{of}\;\text{Eq.}\;\text{(}3\text{)}]-\underset{\text{depletion}\;\text{by}\;\text{anti}-\text{PD}-1}{\underset{︸}{\mu_{PA}PA,}} \end{array} \end{array}$$ (9)

where *μ*_*PA*_ is the depletion rate of PD-1 by anti-PD-1.

PD-L1 from T cells or cancer cells combines with PD-1 on the plasma membrane of T cells to form a complex PD-1-PD-L1 (*Q*_1_) on the T cells [42, 43]. Denoting the association and disassociation rates of *Q*_1_ by *α*_*PL*_ and $d_{Q_{1}}$, respectively, we can write

$$\begin{array}{c} P+L\overset{\alpha_{PL}}{\underset{d_{Q_{1}}}{⇌}}Q_{1}. \end{array}$$

The half-life of *Q*_1_ is less then 1 second (i.e. 1.16 × 10^−5^ day) [44], so that $d_{Q_{1}}$ is very large. Hence we may approximate the dynamical equation for *Q*_1_ by the steady state equation, $\alpha_{PL}PL=d_{Q_{1}}Q_{1}$, or

$$\begin{array}{c} Q_{1}=\sigma_{1}PL, \end{array}$$ (10)

where $\sigma_{1}=\alpha_{PL}/d_{Q_{1}}$. Hence, $K_{TQ_{1}}^{\prime }=\sigma_{1}K_{TQ_{1}}$.

### Equation for TIM-3 (*T*_*M*_), Gal-9 (*G*) and TIM-3-Gal-9 (*Q*_2_)

TIM-3 is expressed on the surface of activated CD8^+^ T cells. If we denote by *ρ*_*M*_ the ratio between the mass of all the TIM-3 proteins in one T cell and the mass of one T cell, then

$$\begin{array}{c} T_{M}=\rho_{M}T. \end{array}$$

The expression of TIM-3 is enhanced by TNF-*α* [32], and further upregulated by anti-PD-1 [24, 32], so that

$$\begin{array}{c} T_{M}=\rho_{M}T[1+\alpha_{T}\frac{T_{\alpha }}{K_{\alpha }+T_{\alpha }}(1+\alpha_{MA}\frac{A}{K_{A}+A})]. \end{array}$$ (11)

Gal-9 is expressed on the surface of cancer cells. We denote by *ρ*_*G*_ the ratio between the mass of all the Gal-9 proteins in one cancer cell and the mass of a cancer cell, so that

$$\begin{array}{c} G=\rho_{G}C. \end{array}$$ (12)

Similarly to Eq (10), we represent the concentration of the complex TIM-3-Gal-9 in the form:

$$\begin{array}{c} Q_{2}=\sigma_{2}T_{M}G, \end{array}$$ (13)

Hence, $K_{TQ_{2}}^{\prime }=\sigma_{2}K_{TQ_{2}}$.

From Eq (11), we see that resistance to anti-PD-1 is due to upregulation of TIM-3 [24], as represented by the parameter *α*_*MA*_, but the level of resistance depends on the concentration of TNF-*α*. This suggests that anti-TNF-*α* will reduce resistance to anti-PD-1.

### Equation for anti-PD-1 (*A*)

We shall consider simple mice experiments where the anti-PD-1 drug is administered in equal amount *γ*_*A*_ at days *t*_1_, *t*_2_, …, and set $\gamma_{A}\left(t\right)=\gamma_{A}\sum_{t_{i}<t}e^{-\beta (t-t_{i})}$ for some *β* > 0. Some of the drug is depleted in the process of blocking PD-1 and some is degraded, or washed out at rate *d*_*A*_. Hence *A* satisfies the following equation:

$$\begin{array}{ccc} \frac{\partial A}{\partial t}-\delta_{A}\nabla^{2}A & = & \gamma_{A}\left(t\right)-\underset{\text{depletion}\;\text{through}\;\text{blocking}\;\text{PD-1}}{\underset{︸}{\mu_{PA}PA}}-\underset{\text{degradation}}{\underset{︸}{d_{A}A.}} \end{array}$$ (14)

### Equation for anti-TNF-*α* (*B*)

Similarly to Eq (14), we take the dynamics of *B* to be:

$$\begin{array}{ccc} \frac{\partial B}{\partial t}-\delta_{B}\nabla^{2}B & = & \gamma_{B}\left(t\right)-\underset{\text{depletion}\;\text{through}\;\text{blocking}\;\text{TNF-}\alpha }{\underset{︸}{\mu_{T_{\alpha }B}T_{\alpha }B}}-\underset{\text{degradation}}{\underset{︸}{d_{B}B,}} \end{array}$$ (15)

where $\gamma_{B}\left(t\right)=\gamma_{B}\sum_{t_{i}^{\prime }<t}e^{-\beta (t-t_{i}^{\prime })}$.

### Equation for cells velocity (u)

As estimated in the section on parameter estimation, the average steady state densities of the immune cells *D*, *T*_8_ and *C* are taken to be (in units of g/cm^3^)

$$\begin{array}{c} \begin{array}{cc} & D=4\times 10^{-4},\;T=1\times 10^{-3},\;C=0.4. \end{array} \end{array}$$ (16)

To be consistent with Eq (1), we take the constant on the RHS of Eq (1) to be 0.4014. We further assume that all cells have approximately the same diffusion coefficient. Adding Eqs (2)–(4), we get

$$\begin{array}{c} 0.4014\times \nabla \cdot u=\sum_{j=2}^{4}[\text{right-hand}\;\text{side}\;\text{of}\;\text{Eq.}\;\text{(j)}]. \end{array}$$ (17)

To simplify the computations, we assume that the tumor is spherical and denote its radius by *r* = *R*(*t*). We also assume that all the densities and concentrations are radially symmetric, that is, they are functions of (*r*, *t*), where 0 ≤ *r* ≤ *R*(*t*). In particular, **u** = *u*(*r*, *t*) **e***r*, where **e**_r_ is the unit radial vector.

### Equation for free boundary (*R*)

We assume that the free boundary *r* = *R*(*t*) moves with the velocity of cells, so that

$$\begin{array}{c} \frac{dR(t)}{dt}=u\left(R\left(t\right),t\right). \end{array}$$ (18)

#### Boundary conditions

We assume that inactive CTL cells that migrated from the lymph nodes into the tumor microenvironment have constant density $\hat{T}$ at the tumor boundary, and, upon crossing the boundary, they are activated by IL-12. We then have the following flux conditions for T cells and *P* at the tumor boundary:

$$\begin{array}{c} \frac{\partial T}{\partial r}+\sigma_{T}\left(I_{12}\right)\left(T-\hat{T}\right)=0,\;\;\text{at}\;\;r=R\left(t\right), \end{array}$$ (19)

$$\begin{array}{c} \frac{\partial P}{\partial r}+\sigma_{T}\left(I_{12}\right)\left(P-\rho_{P}\hat{T}\right)=0,\;\;\text{at}\;\;r=R\left(t\right), \end{array}$$ (20)

where we take $\sigma_{T}\left(I_{12}\right)=\sigma_{0}\frac{I_{12}}{K_{I_{12}}+I_{12}}$. We also assume

$$\begin{array}{c} \begin{array}{cc} \text{no-flux}\;\text{for} & \;D,I_{12},T_{\alpha },A\;\text{and}\;B\;\text{at}\;r=R\left(t\right); \end{array} \end{array}$$ (21)

the boundary condition for *C* is determined by Eq (1).

#### Initial conditions

The initial conditions must be consistent with Eqs (1) and (16), namely,

$$\begin{array}{c} D+T+C=0.4014\;g/{\text{cm}}^{3}, \end{array}$$ (22)

and

$$\begin{array}{c} P=\rho_{P}T\;\;at\;t=0. \end{array}$$ (23)

The last condition ensures that in the control case (where *A* ≡ 0), the function *P* = *ρ*_*P*_ *T* is the solution of Eq (9) with the boundary condition (20). The specific choice of initial conditions for our simulations is

$$\begin{array}{c} \begin{array}{cc} & D_{0}=2\times 10^{-4},\;T_{0}=1\times 10^{-4},C_{0}=0.4011,\;I_{12,0}=1\times 10^{-10},\;T_{\alpha ,0}=1\times 10^{-11},\\ & R(0)=0.5\;\text{cm}\;\text{or}\;R(0)=1\;\text{cm}. \end{array} \end{array}$$ (24)

However, other choices give similar simulation results after a few days.

## Results

**Fig 2:**
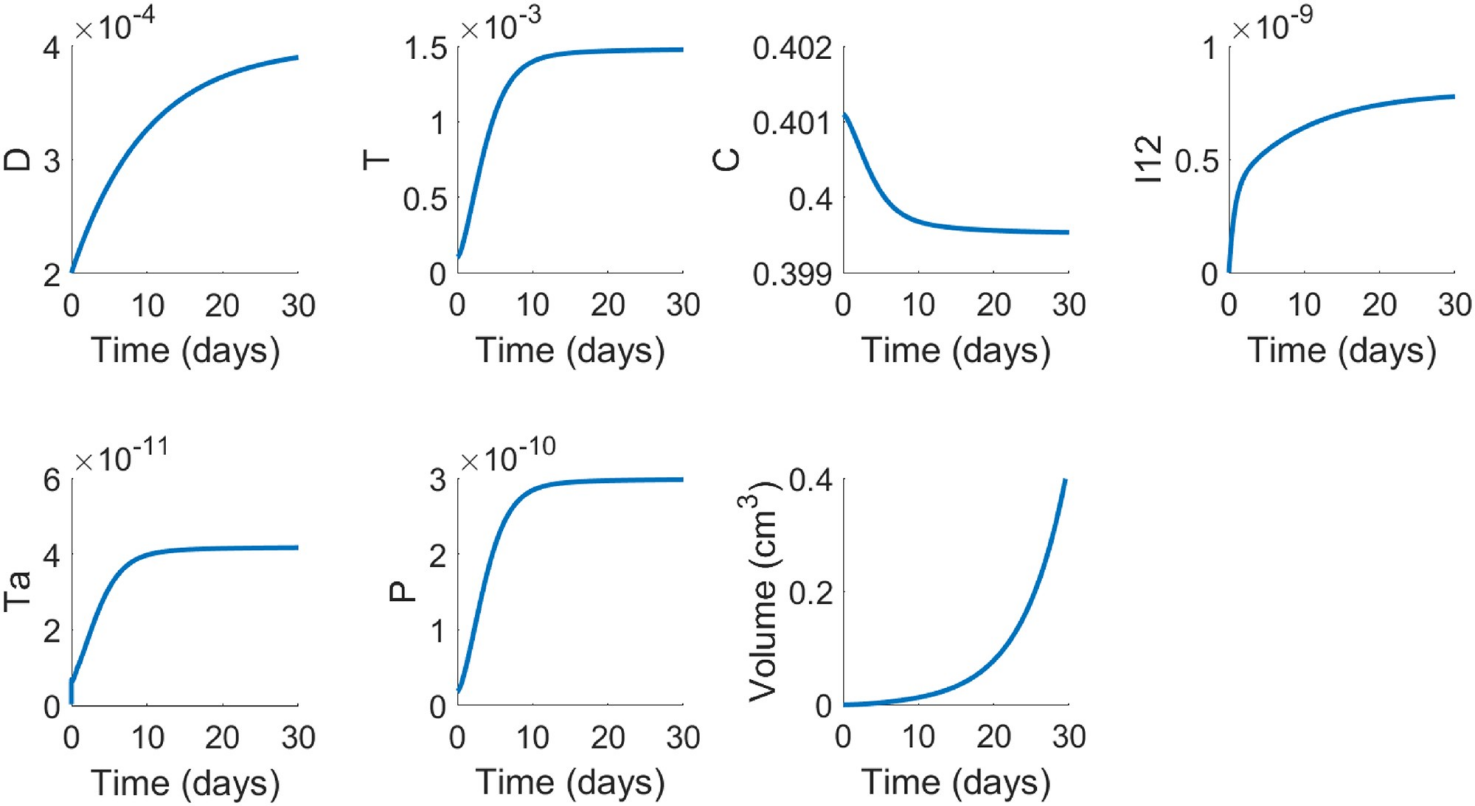
Average densities/concentrations, in g/cm^3^, of all the variables of the model in the control case (no drugs). All parameter values are the same as in Table 2, for the mouse model.

The simulations of the model were performed by Matlab based on the moving mesh method for solving partial differential equations with free boundary [45] (see the section on computational method). Fig 2, in the control case (no drugs), shows the average densities of cells and concentrations of cytokines for the first 30 days, and the growth of tumor volume; We note that the average concentrations of each species, *X*, tends to steady state *X*^0^. In estimating some production parameters (in section of parameter estimation) we made the assumption that each “half-saturation” parameter *K*_*X*_ is equal to *X*^0^, and *X*^0^ is estimated from clinical/experimental data. By comparing the values of *K*_*X*_ in Table 2 with the values *X*^0^ from Fig 2 we see that this assumption is approximately satisfied. Fig 2 shows that, as tumor progresses, the average densities of the anti-cancer immune cells (*D*, *T*) increase, as do the average concentrations of the inflammatory cytokines (*I*_12_, *T*_*α*_), while the tumor volume is increasing exponentially. We can also simulate the spatial variations of the variables, but we are interested, in this paper, only in the average profiles of the variables in order to determine the tumor volume growth (or shrinkage, under treatment). We note however that the diffusion coefficients of cells and cytokines differ by several orders of magnitude, and the boundary condition (19) brings new T cells into the tumor. For these reasons an ODE system cannot adequately represent the dynamics of the tumor volume.

**Table 2:** Summary of parameter values.

Notation	Description	Value used	References
*δ*_*D*_	diffusion coefficient of DCs	8.64 × 10^−7^ cm^2^ day^−1^	[49]
*δ*_*T*_	diffusion coefficient of T cells	8.64 × 10^−7^ cm^2^ day^−1^	[49]
*δ*_*C*_	diffusion coefficient of tumor cells	8.64 × 10^−7^ cm^2^ day^−1^	[49]
$\delta_{I_{12}}$	diffusion coefficient of IL-12	7.17 × 10^−2^ cm^2^ day^−1^	estimated
$\delta_{T_{\alpha }}$	diffusion coefficient of TNF-*α*	8.46 × 10^−2^ cm^2^ day^−1^	estimated
*δ*_*A*_	diffusion coefficient of anti-PD-1	4.76 × 10^−2^ cm^2^ day^−1^	estimated
*δ*_*B*_	diffusion coefficient of anti-TNF-*α*	4.75 × 10^−2^ cm^2^ day^−1^	estimated
*σ*_0_	flux rate of *T* cells at the boundary	1 cm^−1^	[49]
*η*	killing rate of tumor cells by T cells	328.55 cm^3^/g · day	[50]
*μ*_*PA*_	blocking rate of PD-1 by anti-PD-1	1.03 × 10^7^ cm^3^/g · day	estimated
*μ*_*T*_*α*_*B*_	blocking rate of TNF-*α* by anti-TNF-*α*	2.56 × 10^8^ cm^3^/g · day	estimated
*ρ*_*P*_	expression of PD-1 in T cells	2.49 × 10^−7^	[50]
*ρ*_*L*_	expression of PD-L1 in T cells	3.25 × 10^−7^	[50]
*ρ*_*M*_	expression of TIM in T cells	1.5 × 10^−7^	estimated
*ρ*_*G*_	expression of Gal-9 in cancer cells	2 × 10^−8^	estimated
*ε*_*C*_	the ratio of mass of PD-L1 in cancer cell and T cell	0.1	estimated
*α*_*L*_	upregulation rate of PD-L1 by TNF-*α*	1	estimated
*α*_*T*_	upregulation rate of TIM-3 by TNF-*α*	1	estimated
*α*_*MA*_	enhancement of upregulation of TIM-3 by anti-PD-1	2	estimated
$\lambda_{DC}{\hat{D}}_{0}$	activation rate of DCs by tumor cells	8 × 10^−5^ g/cm^3^ · day	[50]
$\lambda_{TI_{12}}{\hat{T}}_{0}$	activation rate of T cells by IL-12	6.48 × 10^−4^ g/cm^3^ · day	estimated
*λ*_*C*_	growth rate of cancer cells in mice	1.295 day^−1^	estimated
*λ*_*C*_	growth rate of cancer cells in humans	0.895 day^−1^	estimated
*λ*_*I*_12_*D*_	production rate of IL-12 by DCs	2.76 × 10^−6^ day^−1^	estimated
*λ*_*T*_*α*_*T*_	production rate of TNF-*α* by T cells	6.48 × 10^−4^ day^−1^	estimated
*d*_*D*_	death rate of DCs	0.1 day^−1^	estimated
*d*_*T*_	death rate of T cells	0.197 day^−1^	estimated
*d*_*C*_	death rate of tumor cells	0.17 day^−1^	[49]
$d_{I_{12}}$	degradation rate of IL-12	1.38 day^−1^	[51]
$d_{T_{\alpha }}$	degradation rate of TNF-*α*	216 day^−1^	[52]
*d*_*A*_	degradation rate of anti-PD-1	0.046 day^−1^	estimated
*d*_*B*_	degradation rate of anti-TNF-*α*	0.069 day^−1^	estimated
*K*_*D*_	half-saturation of dendritic cells	4 × 10^−4^ g/cm^3^	[50]
*K*_*T*_	half-saturation of T cells	1 × 10^−3^ g/cm^3^	[50]
*K*_*C*_	half-saturation of tumor cells	0.4 g/cm^3^	[49]
$K_{I_{12}}$	half-saturation of IL-12	8 × 10^−10^ g/cm^3^	[50, 53]
$K_{T_{\alpha }}$	half-saturation of TNF-*α*	3 × 10^−11^ g/cm^3^	[54]
$K_{TQ_{1}}$	inhibition of function of T cells by PD-1-PD-L1	1.36 × 10^−18^ g^2^/cm^6^	estimated
$K_{TQ_{2}}$	inhibition of function of T cells by Tim-3-Gal-9	1.365 × 10^−18^ g^2^/cm^6^	estimated
*C*_*M*_	carrying capacity of cancer cells	0.8 g/cm^3^	estimated
$\hat{T}$	density of CD8^+^ T cells from lymph node	2 × 10^−3^ g/cm^3^	estimated
*β*	drug control parameter	1.55 g/cm^3^	fitted

Bertrand et al. [32] demonstrated, in a mouse model, that TNF-*α* blockade overcomes resistance to anti-PD-1 in melanoma. In [32] mice were injected with 3 × 10^5^ melanoma cells, and the tumor became detectible by day 6. Drugs (anti-PD-1 and anti-TNF-*α*) were injected at days 6,10,13 in (see Fig 6(d) of [32]) and at days 13, 16, 19 in (see Fig 6(e) of [32]); the tumor volume increase was in the range of 100-350 *mm*^3^.

**Fig 3:**
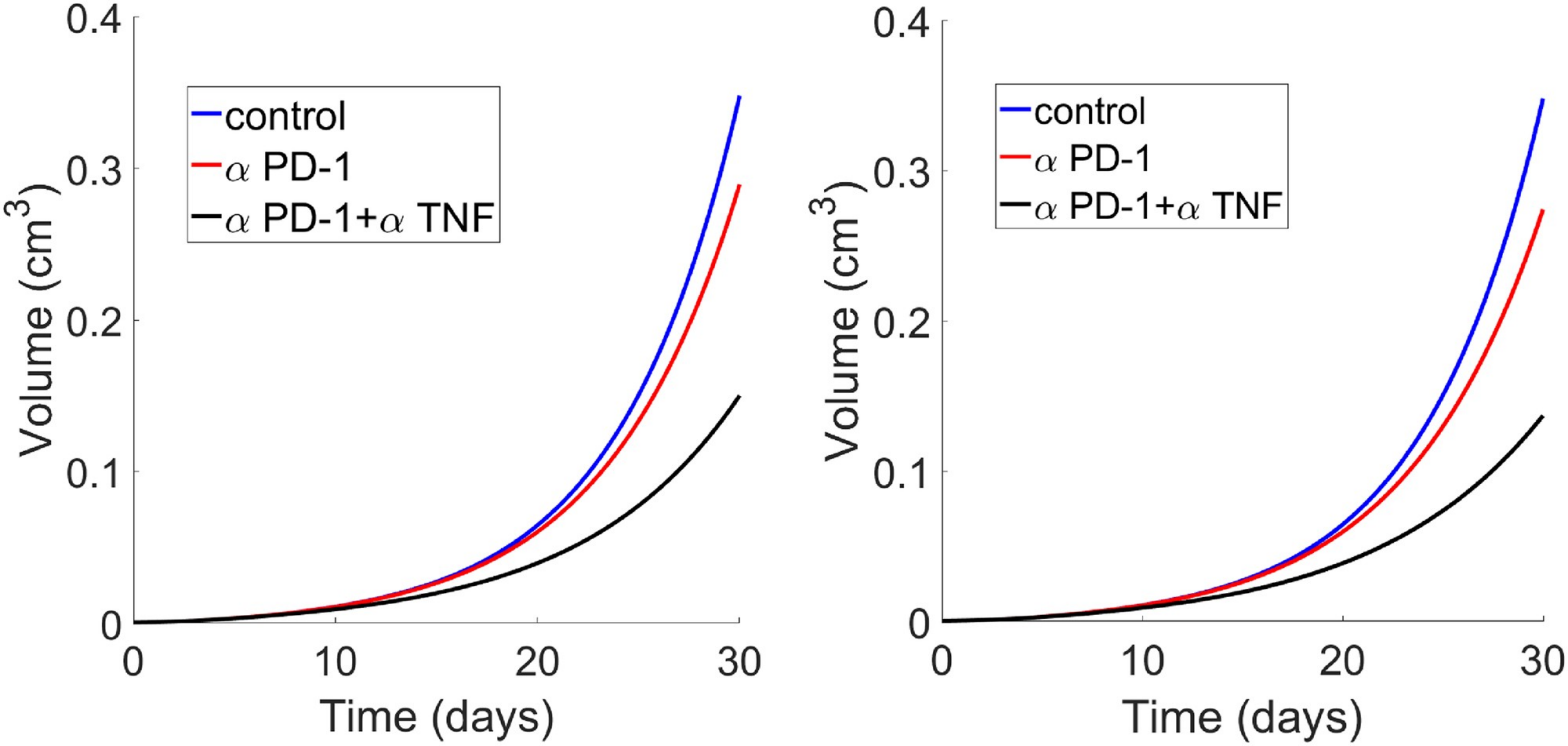
Growth of tumor volume without treatment, or under treatment with *γ*_*A*_, or combination (*γ*_*A*_, *γ*_*B*_). (a) The treatment is at days 6, 10 and 13. (b) The treatment is at days 13, 16 and 19. *γ*_*A*_ = 10^−10^ g/cm^3^ · day, *γ*_*B*_ = 10^−6^ g/cm^3^ · day. All other parameter values are the same as in Table 2, for the mouse model.

Fig 3 shows the growth of tumor volume under treatment with anti-PD-1 (*γ*_*A*_) and anti-TNF-*α* (*γ*_*B*_). Taking *γ*_*A*_ = 10^−10^ g/cm^3^ · day^−1^, *γ*_*B*_ = 10^−6^ g/cm^3^ · day^−1^, and using the same schedules of treatment as in Bertrand et al. [32], Fig 3(a) is in qualitative agreement with Figure 6(d) of Bertrand et al. [32], and Fig 3(b) is in qualitative agreement with Figure 6(e) of [32].

### Clinical trials in silico

We proceed with treatment of humans, given in cycle of 3 weeks. We compare the different scheduling options in a cycle:

- (S1) Both anti-PD-1 (nivolumab) and anti-TNF-*α* (infliximab) are injected in the first day of week 1;
- (S2) Anti-PD-1 is injected in the first day of week 1 and anti-TNF-*α* is injected in the first day of week 2;
- (S3) Anti-TNF-*α* is injected in the first day of week 1 and anti-PD-1 is injected in the first day of week 2.

A course of cancer treatment may take 3 to 8 cycles. As in [46–48] we shall evaluate the efficacy of a treatment by the tumor volume reduction rate (TVRR) at the end-point (final day) of the treatment, where we define

$$\begin{array}{c} \text{TVRR}=\frac{V\left(0\right)-V\left(t_{e}\right)}{V(0)}\times 100\%, \end{array}$$

and *V*(0) = initial tumor volume, *V*(*t*_*e*_) = end-point tumor volume.

**Fig 4:**
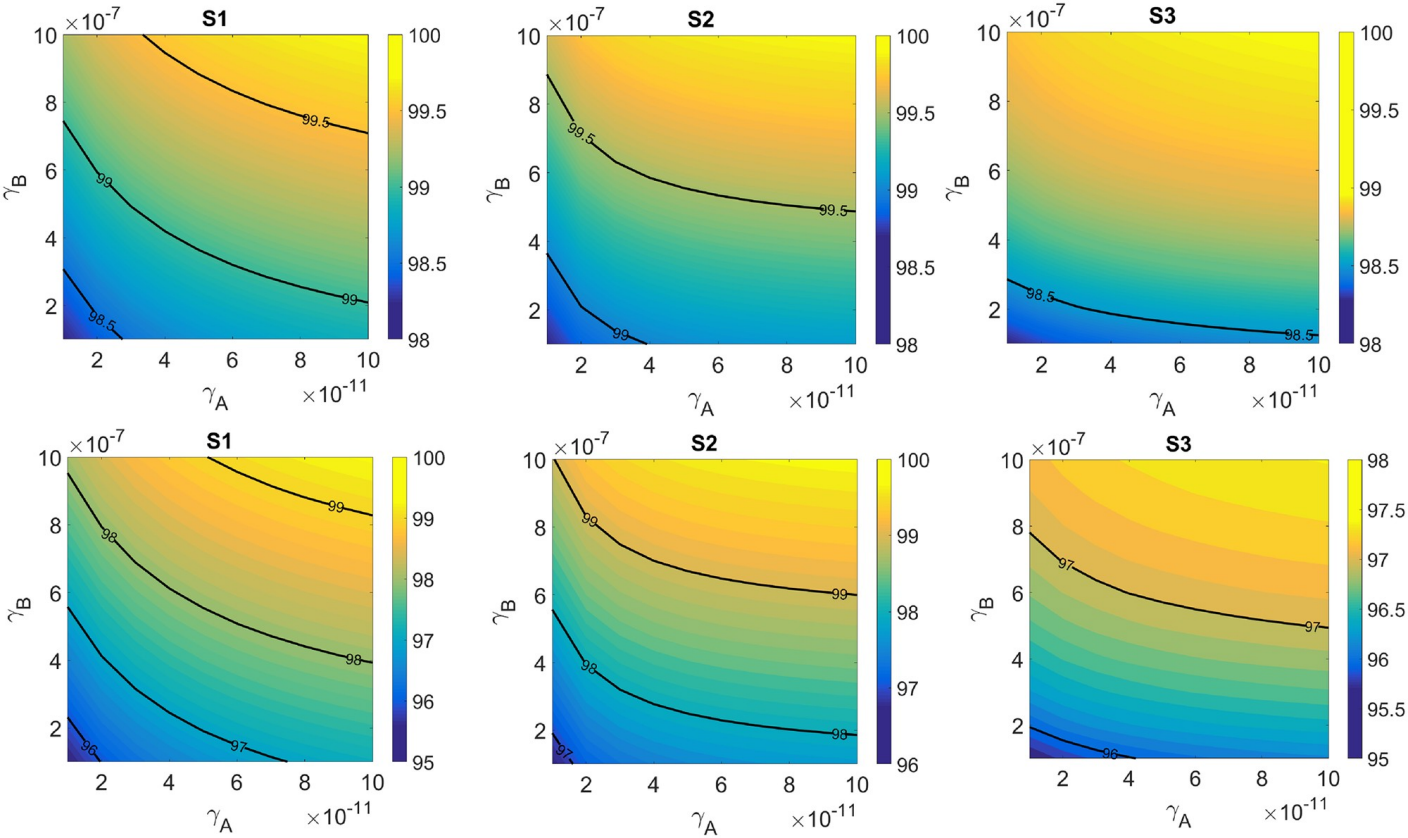
Efficacy map for three schedules: S1 (left), S2 (middle) and S3 (right). The color columns indicate the tumor volume reduction rate (TVRR) after 5 cycles; tumor is initially with the radius *R*(0) = 0.5 cm (upper row) and *R*(0) = 1 cm (lower row).

**Fig 5:**
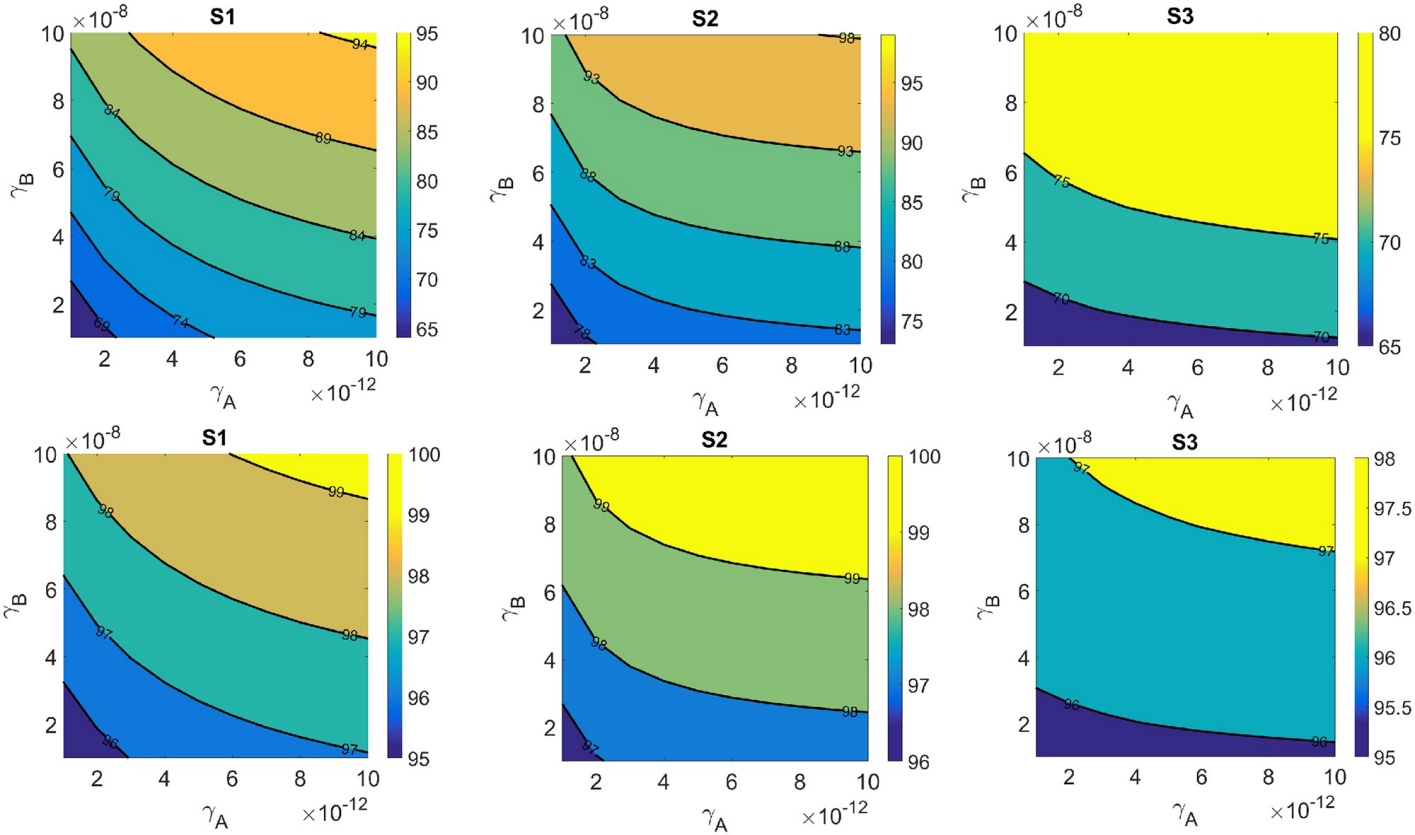
Efficacy map for three schedules: S1 (left), S2 (middle) and S3 (right). The color columns indicate the tumor volume reduction rate (TVRR) after 5 cycles (upper row) and 10 cycles (lower row); tumor is initially with radius *R*(0) = 1 cm.

We note that the doses *γ*_*A*_ and *γ*_*B*_ used in the model are correlated to, but not the same as, the dose amounts used in actual treatments of patients; in particular, the fact that in our model *γ*_*B*_ is several orders of magnitude larger than *γ*_*A*_ does not mean that it is relatively larger than *γ*_*A*_ in clinical treatment. But it is reasonable to expect that the reduction in the doses in the model will have the same effect on the tumor volume growth if the corresponding reduction was made in the treatment of patients. The doses of *γ*_*A*_, *γ*_*B*_ in Fig 3 were chosen in order to fit the simulations with experimental results. For humans we took a smaller tumor cells growth, and smaller ranges of *γ*_*A*_, *γ*_*B*_ in Figs 4 and 5 (below) in order to get the reduction of tumor volume to be in the same range as in clinical data; In [47], under two 3-week cycles in treatment of breast cancer, TVRR was >65%, and in [48], under four 2-week cycles treatment of rectal cancer, TVRR median was 52% but, for significant number of patients, it was >65%.

In Fig 4 we simulated the TVRR for a tumor with initial radius *R*(0) = 0.5 cm (volume 0.5236 cm^3^), in the upper row, and *R*(0) = 1 cm (volume 3.1456 cm^3^) in the lower row, under 5 cycle treatment course, undergoing 5 cycles with doses of *γ*_*A*_ and *γ*_*B*_ in the ranges of 0.1 × 10^−11^−1 × 10^−11^ g/cm^3^ · day and 0.1 × 10^−6^−1 × 10^−6^ g/cm^3^ · day, respectively. We see that, in the treatment of both tumors, S2 is somewhat more effective than S1, while S1 is significantly more effective than S3. We can explain this as follows:

Under S1, resistance to anti-PD-1 begins at day 1 of a cycle, but its effect may be still small in the first few days. So it is more effective to apply the resistance-blocking drug (*γ*_*B*_) in the first day of week 2 of a cycle rather than immediately in day 1 of week 1. On the other hand, under schedule S3, blocking anti-PD-1 resistance when *γ*_*A*_ is injected in day 1 of the second week of a cycle will occur only 2 weeks later when *γ*_*B*_ is injected in day 1 of week 1 of the following cycle. Hence the blockade of resistance comes “too late” and is not very effective. We conclude that, in order to overcome the resistance to anti-PD-1 drug (*γ*_*A*_) most effectively, the anti-TNF-*α* drug (*γ*_*B*_) should be injected not too soon but definitely not too late after the injection of *γ*_*A*_.

In Fig 5 we repeated the simulations of Fig 4 with *R*(0) = 1 cm and a smaller range of doses; we did it for one treatment with 5 cycles and an extended treatment with 10 cycles. We first see that, once again, S2 is somewhat more effective than S1, and S1 is significantly more effective than S3. But we can also see from Fig 5 the benefits of extended treatment. For example, under schedule S2, with *R*(0) = 1 cm, the TVRR will increase from 75%, 85% and 90% to 96%, 97.8% and 99%, respectively, if the treatment is extended from 5 cycles to 10 cycles. By comparing the treatments with different ranges of dose, in the case of schedule S2 with *R*(0) = 1 cm, we see that if the range is decreased by a factor of 1/10 then the TVRR will decrease, for instances, from 98–99% to 82–85%. We also see from Fig 4 that under the sane treatment, the TVRR is larger for the tumor with the smaller initial volume. This can be explained by the fact that concentration of the same dose injected into a small tumor is larger than that injected into a larger tumor.

## Conclusion

Drug resistance is a major obstacle to cancer treatment. Patients respond well for several months, or even years, but relapse eventually occurs. This is the case with both chemotherapy and immune therapy drugs. Anti-Cancer T cells have ‘brakes’ (called checkpoints) on their activities in the form of membranous proteins such as PD-1 and TIM-3; when PD-L1 (or Gal-9) combines with PD-1 (or TIM-3) the T cell activation is inhibited. A drug, anti-PD-1, is increasing used in the treatment of cancer, and in the present paper we considered the problem of how to deal with cancer resistance to anti-PD-1. We focused on the role of TNF-*α* in reducing this resistance.

TNF-*α* increases the production of PD-L1, which renders anti-PD-1 less effective. TNF-*α* also activates expression of TIM-3, which enables Gal-3, expressed on cancer cells, to block T cells by combing with TIM-3 on T cells membrane. These facts suggest that anti-TNF-*α* could be an effective drug in reducing resistance to anti-PD-1.

We developed a mathematical model where anti-PD-1 is combined with anti-TNF-*α*. The model is represented by a system of PDEs. The model is ‘minimal’ in the sense that it includes only cancer cells, T cells and dendritic cells, and the most relevant protein populations needed to address the effectiveness of the combined therapy. Simulations of the model were shown to be in agreement with mice experiments.

The model can be used to assess the efficacy of various protocols of treatment with the combination of the two drugs. As an example, we considered the following scheduling cycles.: Both drugs administered in day 1 of week 1 (S1), anti-PD-1 injected in day 1 of week 1 and anti-TNF-*α* injected in day 1 of week 2 (S2), or in the inverse order (S3). For each pair of (anti-PD-1, anti-TNF-*α*) in some range we marked on the color columns the tumor volume reduction rate (TVRR) at the end of the treatment. In Figs 4 and 5 it was shown that schedule S2 is somewhat more beneficial than schedule S1, while schedule S1 is significantly more beneficial than schedule S3; these results mean that in order for the blockade of the drug resistance to anti-PD-1, anti-TNF-*α* should not be injected too late after the injection of anti-PD-1, but also not too early. This result should be viewed as an hypothesis, to be validated in clinical trials.

Figs 4 and 5 also show how TVRR increases as the dose level increases when the range of doses increases by a factor of 10, or when the treatment, with the same dose range, is given to a tumor with a smaller volume.

The model developed in this paper can be used also be explore the efficacy of schedules with shorter cycles when such clinical trials become more prevalent.

## Supplementary materials

### Computational method

We consider the following convection-diffusion equation to illustrate our computational method:

$$\begin{array}{c} \frac{\partial X}{\partial t}+div\left(vX\right)=D\nabla^{2}X+F_{X}, \end{array}$$ (25)

where *F*_*X*_ accounts for all the right-hand side terms. Since the model we consider is a free boundary problem, we employ the moving mesh method to compute it. We write Eq (25) can be written in the total derivative form

$$\begin{array}{c} \frac{dX(r(t),t)}{dt}+div\left(v\right)X=D\nabla^{2}X\left(r\left(t\right),t\right)+F_{X}. \end{array}$$

Let $r_{i}^{n}$, $X_{i}^{n}$ denote numerical approximations of i-th grid point and $X(r_{i}^{n},t)$, respectively, when *t* = *nτ*, where *τ* is the time stepsize. The discretization is derived by the explicit Euler finite difference scheme, i.e.,

$$\begin{array}{c} \frac{X_{i}^{n+1}-X_{i}^{n}}{\tau }+(\frac{v_{r}}{r_{i}^{n}}+v_{i}^{n})X_{i}^{n}=D(X_{rr}+\frac{X_{r}}{r_{i}^{n}})+F_{X}, \end{array}$$

where $X_{r}=\frac{h_{-1}^{2}X_{i+1}^{n}-h_{1}^{2}X_{i-1}^{n}-\left(h_{1}^{2}-h_{-1}^{2}\right)X_{i}^{n}}{h_{1}\left(h_{-1}^{2}-h_{1}h_{-1}\right)}$, $X_{rr}=2\frac{h_{-1}X_{i+1}^{n}-h_{1}X_{i-1}^{n}+\left(h_{1}-h_{-1}\right)X_{i}^{n}}{h_{1}\left(h_{1}h_{-1}-h_{-1}^{2}\right)}$, and $h_{-1}=r_{i-1}^{n}-r_{i}^{n}$, $h_{1}=r_{i+1}^{n}-r_{i}^{n}$. Then the mesh is moving by $r_{i}^{n+1}=r_{i}^{n}+v_{i}^{n}\tau$, where $v_{i}^{n}$ is solved by the velocity equation. In order to make the Euler method stable, we take $\tau \leq \frac{min\{h_{1},h_{-1}\}}{2D}$.

### Parameter estimation

#### Half-saturation

In an expression $Y\frac{X}{K_{X}+X}$ which represents an activation of a species *Y* by a species *X*, the parameter *K*_*X*_ is called the “half-saturation” of *X*. We assume that the average density/concentration of each species, *X*, tends to steady state *X*^0^, and that the quotient *X*^0^/(*K*_*X*_ + *X*^0^) is not too close to 0 or to 1, and take

$$\begin{array}{c} \frac{X^{0}}{K_{X}+X^{0}}=\frac{1}{2},\;\;\text{or}\;\;K_{X}=X^{0}. \end{array}$$ (26)

The values *X*^0^ will be estimated from clinical/experimental data, and then the *K*_*X*_ are determined by (26).

#### Diffusion coefficients

Young [55] established the following formula for the diffusion coefficient, *D*_*p*_, of any protein *p*:

$$\begin{array}{c} D_{p}=\frac{A}{M_{p}^{1/3}}, \end{array}$$ (27)

where *M*_*p*_ is the molecular weight of *p* and *A* is a constant. In particular

$$\begin{array}{c} D_{p}=\frac{M_{V}^{1/3}}{M_{p}^{1/3}}D_{V}, \end{array}$$ (28)

where *M*_*V*_ and *D*_*V*_ are respectively the molecular weight and diffusion coefficient of VEGF: *M*_*V*_ = 24kDa [56] and *D*_*V*_ = 8.64 × 10^−2^ cm^−2^d^−1^ [57]. The following table lists the molecular weight of the proteins in our model, taken from [56], and their corresponding diffusion coefficients computed from the formula (28): The molecular weights of nivalumab (*A*) and infliximab (*B*) are 143.6 kDa and 144.2 kDa, respectively. Hence, by formula (28), we get *D*_A_ = 4.76 × 10^−2^ cm^2^d^−1^ and *D*_B_ = 4.75 × 10^−2^ cm^2^d^−1^.

**Table:** 

*X*	*I*_12_	*T*_*α*_
Molecular Weight(kDa)	37.2	25.6
*D*_*X*_ (10^−2^ cm^2^d^−1^)	7.17	8.46

#### Diffusion coefficients of cells

For simplicity, we assume that all cell types have the same diffusion coefficient, and as in [57], we take

$$\begin{array}{c} D_{X}=8.64\times 10^{-7}\;{\text{cm}}^{2}d^{-1}, \end{array}$$

#### Death/Degradation rates

From [58] we take *d*_*T*_ = 0.18/d, and from [49] we take *d*_*D*_ = 0.1/d and *d*_*C*_ = 0.17/d. The half-life of *I*_12_ in peripheral blood is 352 minutes and in chord blood 614 minutes [51]. We accordingly take the half-life of *I*_12_ in tissue to be 5 hours, so that $d_{I_{12}}=1.38/\text{d}$. The half-life of TNF-*α* is 4.6 minutes [52]; hence, $d_{T_{\alpha }}=216/\text{d}$.

#### Inhibition of *T* by *Q*_1_, *Q*_2_

From the notation $K_{TQ_{1}}^{\prime }=\sigma_{1}K_{TQ}$, we deduce, as in [59], that $K_{TQ_{1}}=1.36\times 10^{-18}\text{g}/{\text{cm}}^{3}$. We assume a similar inhibition parameter for the checkpoint *T*_*M*_, taking $K_{TQ_{2}}=1.365\times 10^{-18}\text{g}/{\text{cm}}^{3}$.

#### Production parameters

In order to estimate production parameters, we use the “steady-state” of each equation, that is, we equate the right-hand side of each equation to zero, replace each species *X* in this equation by *X*^0^ and each *K*_*X*_ by *X*^0^.

Eq (2). In steady state, we have

$$\begin{array}{c} \lambda_{DC}\frac{{\hat{D}}_{0}}{2}=d_{D}D^{0}, \end{array}$$

The number of dendritic cells in advanced melanoma can increase to 400 cells/*mm*^3^ [60]. Taking it to be 200 cells/*mm*^3^ and assuming that the mass of one DC is 5 × 10^−10^ g, we get *D*^0^ = 4 × 10^−4^. Since *d*_*D*_ = 0.1/day [49], we obtain

$$\begin{array}{c} \lambda_{DC}{\hat{D}}_{0}=2d_{D}K_{D}=8\times 10^{-5}. \end{array}$$

Eq (3). we assume that in steady state,

$$\begin{array}{c} \frac{1}{1+PL/K_{TQ_{1}}}\frac{1}{1+T_{M}G/K_{TQ_{2}}}=\frac{1}{1.8} \end{array}$$

Hence, in steady state of Eq (3),

$$\begin{array}{c} \lambda_{TI_{12}}{\hat{T}}_{0}\cdot \frac{1}{2}\cdot \frac{1}{1.8}-d_{T}T^{0}=0. \end{array}$$

The number of CD8^+^ T cells in melanoma is in the range of 7 − 10 × 10^5^ cells/*cm*^3^ [61]. Taking the mass of one cells to be 5 × 10^−10^, we estimate the density of CD8^+^ T cells at 5 × 10^−4^ *g*/*cm*^3^. Since T includes Th1 cells, we take *K*_*T*_ = *T*^0^ = 10^−3^ *g*/*cm*^3^. Since *d*_*T*_ = 0.18/day [58], we get $\lambda_{TI_{12}}{\hat{T}}_{0}=6.48\times 10^{-4}$ *g*/*cm*^3^/day.

Eq (4). We take, in steady state, *C* = 0.4 g/cm^3^ [49] and *C*_*M*_ = 0.8 g/cm^3^, *η* = 328 0.8 g/cm^3^/g · day^−1^ [50], *d*_*C*_ = 0.17/day [58]. Hence, in steady state,

$$\begin{array}{c} \lambda_{C}\cdot \frac{1}{2}-\eta K_{T}-d_{C}=0, \end{array}$$

so that *λ*_*C*_ = 0.996/day. Since, however, the tumor volume is increasing on account of the abnormal proliferation of cancer cells, we increase this value by a factor, taking *λ*_*C*_ = 1.2×0.996 = 1.295/day. This value will be used in mouse models, as in Figs 2 and 3. But for humans (Figs 4 and 5) it is assumed that the tumor cells proliferation is slower, taking *λ*_*C*_ = 0.885/day.

Eq (5). From the steady state equation we get

$$\begin{array}{c} \lambda_{I_{12}D}K_{D}-d_{I_{12}}K_{I_{12}}=0. \end{array}$$

Since *K*_*D*_ = 4 × 10^−4^ g/cm^3^, $K_{I_{12}}=8\times 10^{-10}\text{g}/{\text{cm}}^{3}$ [50, 53], and $d_{I_{12}}=1.38/\text{day}$ [51], we get *λ*_*I*_12_*D*_ = 2.76 × 10^−6^/day.

Eq (6). From the steady state equation in the control case we get

$$\begin{array}{c} A_{T_{\alpha }}+\lambda_{T_{\alpha }T}K_{T}-d_{T_{\alpha }}K_{T_{\alpha }}=0. \end{array}$$

We take $A_{T_{\alpha }}=1.12\times 10^{-9}$ *g*/*cm*^3^day. Since $K_{T_{\alpha }}=3\times 10^{-11}\text{g}/{\text{cm}}^{3}$ [54] and $d_{T_{\alpha }}=216/\text{day}$ [52], we get *λ*_*T*_*α*_*T*_ = 1.08 × 10^−6^/day.

**Remaining parameters.** We take *ρ*_*P*_ = 2.49 × 10^−7^ [50]. Then, in steady state with *T* = *K*_*T*_ = 10^−3^ g/cm^3^ and *P* = 2.49 × 10^−10^ g/cm^3^. We take *ρ*_*L*_ = 3.25 × 10^−7^ g/cm^3^ [50] and assume that the expression of PD-L1 in cancer cells is less than it is on T cells, taking *ε*_*C*_ = 0.1 in Eq (8). We also take *α*_*L*_ = 1 which means that TNF-*α* increases the expression of PD-L1 by 50%, in steady state.

We assume that TIM-3 is expressed on T cells at a lower level than PD-1, that is, *ρ*_*M*_ < *ρ*_*P*_, and take

$$\begin{array}{c} \rho_{M}=1.5\times 10^{-7}. \end{array}$$

We also take (in Eq (11)) *α*_*T*_ = 1 and *α*_*MA*_ = 2. We similarly assume that Gal-9 is expressed in cancer cells at a lower level than PD-L1 (in Eq (8)), that is, *ρ*_*G*_ < *ρ*_*L*_ *ε*_*C*_, and take *ρ*_*G*_ = 2 × 10^−8^.

The half-life od nivolumab is 10-20 days. Taking it to be 15 days, we get

$$\begin{array}{c} d_{A}=\frac{\text{ln}2}{15}=0.046\;{\text{day}}^{-1}. \end{array}$$

The half-life of infliximab is 7-12 days. Taking half-life of 10 days, we get

$$\begin{array}{c} d_{B}=\frac{\text{ln}2}{10}=0.069\;{\text{day}}^{-1}. \end{array}$$

We assume that 10% of *A* is used in blocking PD-L1 while the remaining 90% degrades naturally or decreased by washout, that is

$$\begin{array}{c} \mu_{PA}PA/10\%=d_{A}A/90\%. \end{array}$$

Recalling that, in steady state, with *P* = 2.49 × 10^−10^, g/cm^3^, we get

$$\begin{array}{c} \mu_{PA}=\frac{0.046}{9\times 2.49\times 10^{-10}}=1.03\times 10^{7}\;{\text{cm}}^{3}/g\cdot \text{day}. \end{array}$$

Under a similar assumption on the depletion of *B*, in steady state

$$\begin{array}{c} \mu_{T_{\alpha }B}=\frac{d_{B}}{9K_{T_{\alpha }}}=\frac{0.069}{9\times 3\times 10^{-11}}=2.56\times 10^{8}\;{\text{cm}}^{3}/g\cdot \text{day}. \end{array}$$

### Sensitivity analysis

**Fig 6:**
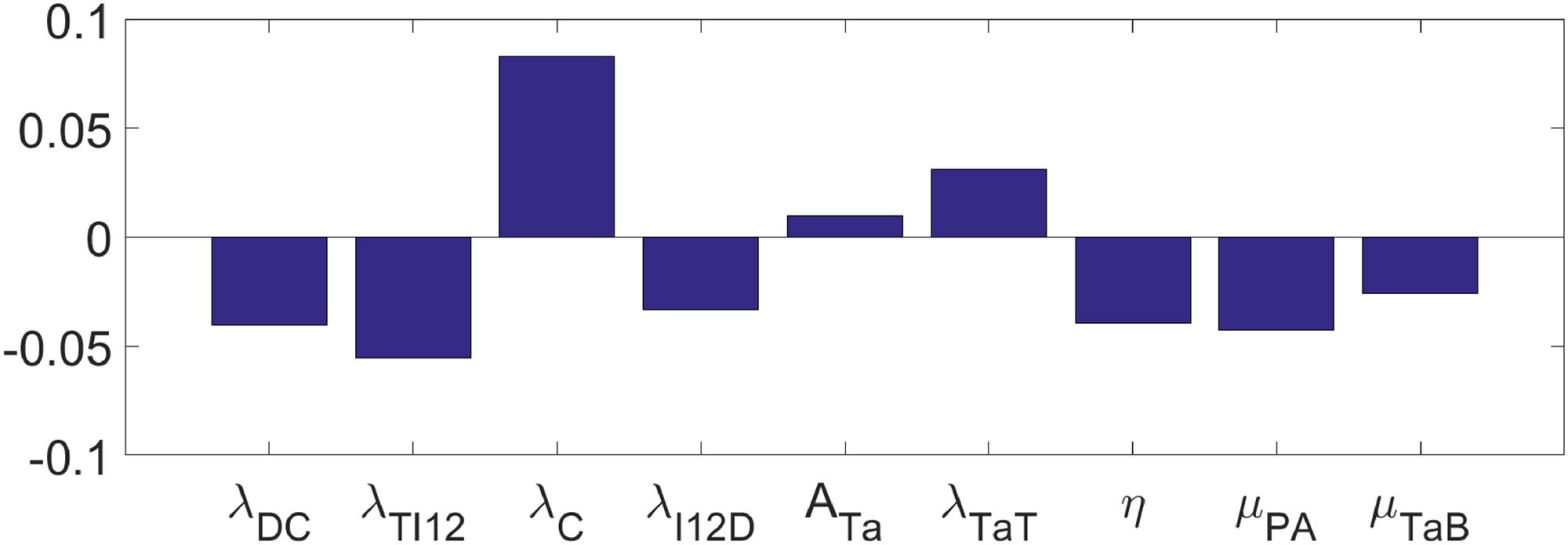
Sensitivity analysis of the PRCC values with respect to the radius of the tumor.

We performed sensitivity analysis on the production parameters in Eqs (2)–(6), the depletion rates $\mu_{BT_{\alpha }}$ and *μ*_*PA*_ in Eqs (6) and (9), and *η* in Eq (4). Following the method in [62], we performed Latin hypercube sampling and generated 1000 samples to calculate the partial rank correlation (PRCC) and the p-values with respect to the radius of the tumor at day 30. The results are shown in Fig 6 (The p-value was <0.01). Note that as *T*_*α*_ increases, cancer increases. Hence $A_{T_{\alpha }}$, *λ*_*T*_*α*_*T*_ are positively correlated and *μ*_*T*_*α*_*B*_ is negatively correlated. On the other hand, as *T* increases, cancer decreases. Hence *λ*_*DC*_, $\lambda_{TI_{12}}$, *λ*_*I*_12_*D*_ and *μ*_*PA*_ (in Fig 6) are negatively correlated. Finally the killing rate *η* of *C* by *T* is negatively correlated while the growth rate *λ*_*C*_ of *C* is highly positively correlated.
